# Genome editing of *SlMYB3R3*, a cell cycle transcription factor gene of tomato, induces elongated fruit shape

**DOI:** 10.1093/jxb/erac352

**Published:** 2022-09-07

**Authors:** Qingyou Zheng, Rie Takei-Hoshi, Hitomi Okumura, Masaki Ito, Kohei Kawaguchi, Shungo Otagaki, Shogo Matsumoto, Zhengrong Luo, Qinglin Zhang, Katsuhiro Shiratake

**Affiliations:** Graduate School of Bioagricultural Sciences, Nagoya University, Nagoya 464-8601, Japan; Key Laboratory of Horticultural Plant Biology, Huazhong Agricultural University, Wuhan 430070, China; Graduate School of Bioagricultural Sciences, Nagoya University, Nagoya 464-8601, Japan; Graduate School of Bioagricultural Sciences, Nagoya University, Nagoya 464-8601, Japan; School of Biological Science and Technology, College of Science and Engineering, Kanazawa University, Kanazawa 920-1192, Japan; Graduate School of Bioagricultural Sciences, Nagoya University, Nagoya 464-8601, Japan; Graduate School of Bioagricultural Sciences, Nagoya University, Nagoya 464-8601, Japan; Graduate School of Bioagricultural Sciences, Nagoya University, Nagoya 464-8601, Japan; Key Laboratory of Horticultural Plant Biology, Huazhong Agricultural University, Wuhan 430070, China; Key Laboratory of Horticultural Plant Biology, Huazhong Agricultural University, Wuhan 430070, China; Graduate School of Bioagricultural Sciences, Nagoya University, Nagoya 464-8601, Japan; University of Trento, Italy

**Keywords:** Cell cycle, CRISPR/Cas9, cytokinesis, fruit shape, MSA motif, MYB3R, transcription factor, tomato

## Abstract

Fruit shape is an important trait that attracts consumers, and the regulation of genes related to cell division is crucial for shaping multicellular organs. In Arabidopsis, MYB3R transcription factors, which harbor three imperfect repeats in the N-terminus, control organ growth by regulating cell division. However, the function of MYB3Rs in tomato remains unknown. Here, we characterized tomato *SlMYB3R3*, which was preferentially expressed in flowers and placed in a subclade with two Arabidopsis cell cycle suppressors (*MYB3R3/5*)*. slmyb3r3* knockout mutants were generated using the CRISPR/Cas9 system. Morphological observation of the *slmyb3r3* mutants showed that fruits that were elongated and occasionally peanut-like in shape were formed, which was caused by significantly increased cell numbers in the longitudinal direction. Transcriptome and yeast one-hybrid assay results suggested that *SlMYB3R3* acted as a suppressor of cell-cycle-related genes by binding to the mitosis-specific activator (MSA) motifs in their promoters. Taken together, knock out of the suppressor *SlMYB3R3* leads to elongated fruit, which results from the altered cell division pattern at the ovary stage, by regulating cell-cycle-related genes in an MSA-dependent manner. Our results suggest that *SlMYB3R3* and its orthologs have the potential to change fruit shape as part of the molecular breeding of fruit crops.

## Teaser

Knock out of *SlMYB3R3*alters the cell division pattern in tomato ovary and leads to elongated fruits by regulating cell-cycle-related genes at the ovary stage in an MSA-dependent manner.

## Introduction

Tomato (*Solanum lycopersicum* L.) is a critically important crop bearing fleshy fruits that are a crucial source of essential nutrients in the daily diet of humans. The growth of tomato fruits primarily relies on the fine regulation of cell number and size, which are determined by the cell cycle process. The cell cycle comprises two main stages: interphase (further subdivided into the G1, S, and G2 phases) and mitosis (M). Entry into or exit from each phase of the cell cycle is influenced by internal (genotypic) and external (environmental) cues. Some *MYB* genes play central roles in genetic control. The MYB superfamily contains more than 100 members with various DNA-binding domain repeats (up to four) at the N-terminus. Among them, R2R3-type MYBs constitute the largest subclade within the MYB superfamily and serve diverse functions in plant biological processes, such as organ development, resistance response, signal transduction, and secondary metabolite biosynthesis (Dubos *et al.*, 2010). In contrast, R1R2R3-type MYB (hereafter designated as MYB3R) transcription factors (TFs) constitute a small class of the MYB superfamily with only five members in Arabidopsis (*Arabidopsis thaliana*) and tobacco (*Nicotiana tabacum*), respectively. MYB3Rs serve evolutionarily conserved functions in cell cycle regulation, especially in controlling G2/M-phase-specific genes such as cyclins (*CYCs*) and cyclin-dependent kinases (*CDKs*) (Ito, 2005; Inzé and De Veylder, 2006). Overexpression of the tobacco *MYB3R* genes *NtmybA1* and *NtmybA2* in BY2 cells activates the expression of genes specific to the G2/M phases, such as *CYCB1*, the cytokinesis-related *NACK1* and *KNOLLE* genes, and some uncharacterized kinesins. The expression of these genes drives cells to the M phase. In contrast, the MYB3R gene *NtmybB* suppresses these genes. All three MYB3Rs function by binding to the mitosis-specific activator (MSA) motifs upstream of G2/M-phase genes with the core sequence 5ʹ-AACGG-3ʹ (Ito *et al*., 1998, 2001; Kato *et al.*, 2009). Arabidopsis *MYB3R1* and *MYB3R4* are orthologous to the transcriptional activators *NtmybA1* and *NtmybA2*. Arabidopsis *myb3r1*/*4* double mutants showed decreased expression of a subset of G2/M-specific genes, namely *CYCA1*, *CYCB1*, *CDKB2*, *CYCB2*, *CDC20.1*, and *KNOLLE*, whose promoters show significant overrepresentation of MSA motifs. These *myb3r1*/*4* double mutants also showed abnormal seedling development, with fused cotyledons, short hypocotyls and roots, reduced internode length and plant stature, and abnormal seed morphology. In addition, an irregular cell division plane was observed early in embryo development, leading to an aberrant embryo shape (Haga *et al*., 2007, 2011). *MYB3R3/MYB3R5* are repressor-type regulators of the cell cycle in Arabidopsis and work redundantly to repress G2/M-specific genes and microtubule-associated proteins. Longer roots and enlarged leaves and seeds were observed in *myb3r1/3/5* mutants of Arabidopsis. Similar to the *myb3r1*/*4* double mutants, the orientation of cell division is disturbed in the *myb3r1/3/5* mutants (Kobayashi *et al.*, 2015; Chen *et al.*, 2017). Plant-specific B-type CDKs (*CDKB*s) reach maximal expression during the G2/M phases under the regulation of MYB3Rs (Inzé and De Veylder, 2006; De Veylder *et al.*, 2007). Overexpression of *CDKB1/2* driven by a fruit-specific promoter leads to irregular and smaller fruit with reduced cell layers and sizes in the pericarp. Knock down of *CDKA1* results in phenotypes similar to those of *CDKB* overexpression (Czerednik *et al.*, 2012). Overexpression of the B-type cyclin *SlCycB2* in tomato leads to abnormal development of floral organs, decreased trichome density on stems and leaves, and decreased terpene content (Gao *et al.*, 2017). These cell-cycle-related genes are closely associated with organ outgrowth.

Fruit shape has become an important goal in breeding of tomatoes for various culinary purposes. Tomato fruit can have various shapes, which can be classified into eight categories: flat, rectangular, ellipsoid, obovoid, round, long, oxheart, and heart-shaped (Paran and van der Knaap, 2007; Rodríguez *et al.*, 2011). Many genes related to variations in fruit shape have been identified in tomato. Obovoid (pear-like) shape is regulated by the gene *OVATE*, which encodes a member of the OVATE family of proteins, which represses fruit elongation, thereby controlling the growth of pear-shaped fruit. The enormously elongated end in the proximal part of the fruit is largely ascribed to increased cell proliferation in this region (Liu *et al.*, 2002; Wu *et al.*, 2018). The gene *SUN* regulates the elongated shape of tomato fruit (Xiao *et al.*, 2008). SUN is a calmodulin-binding protein in the IQ67-domain (IQD) family. Overexpression of *SUN* in tomato confers the elongated fruit shape via alteration of cell division, with higher cell numbers in the longitudinal direction and lower cell numbers in the transverse direction (Xiao *et al.*, 2008; Wu *et al.*, 2011). Fine-tuned division and expansion of cells are the two major principles of regulation of organ morphogenesis (van der Knaap *et al.*, 2014; van der Knaap and Østergaard, 2018). However, the effects of *MYB3R*s on cell cycle regulation during tomato growth remain unclear.

In the present study, we identified four *MYB3R* genes in the tomato genome. *SlMYB3R3* was placed in a subclade with two Arabidopsis cell cycle suppressor genes, *MYB3R3*/5, and was highly expressed in tomato flowers. We characterized the functions of *SlMYB3R3* by developing *slmyb3r3* mutants using the CRISPR/Cas9 system. The *slmyb3r3* mutants exhibited abnormal reproductive organ development, with elongated and occasional peanut-like fruit shapes, which was achieved by altering cell division. These results demonstrate that *SlMYB3R3* is a novel fruit shape regulator in tomato that regulates specific fruit shape outgrowth by directly targeting cell-cycle-related genes, especially cytokinesis-related genes.

## Materials and methods

### Plant materials

Tomato *S. lycopersicum* ‘Suzukoma’ was used as the wild type (WT). The *slmyb3r3* knockout mutants were produced by the CRISPR/Cas9 system. Plants of ‘Suzukoma’, its *slmyb3r3* mutants, ‘Radana’, and ‘San Marzano’ were sown on vermiculite and grown for 7 d in a growth chamber at 25 °C under 16 h light/8 h dark. Then, the seedlings were transferred to peat soil (Hokkaido PeatMoss, Japan) and grown in the glasshouse at Nagoya University (Nagoya, Japan). The plants were watered with water containing the fertilizers Otsuka House No. 1 and No. 2 (Otsuka Chemical, Japan) every day.

### Identification of tomato SlMYB3Rs, multiple sequence alignment, and phylogenetic analysis

Tomato SlMYB3Rs were identified from International Tomato Annotation Group release 3.2 (ITAG 3.20) predicted proteins registered in the Sol Genomics Network (https://solgenomics.net/) by BLASTp using the Arabidopsis MYB3Rs as queries. Sequences of MYB3Rs of other plants were obtained from NCBI (https://www.ncbi.nlm.nih.gov/) or Phytozome (https://phytozome.jgi.doe.gov/pz/portal.html); their accession numbers are listed below. Amino acid sequence alignments were produced by ClustalX software, and conserved domains were defined by using SMART (http://smart.embl-heidelberg.de/). The phylogenetic tree was constructed by MEGA (v.5.0) using the neighbor-joining method with 1000 bootstrap replicates.

### Quantitative real-time PCR analyses

The various organs or tissues were harvested from WT plants. Flowers (with sepals, petals, fused stamen cones, and pistils, excluding the ovary) were sampled at the anthesis stage; the ovary was collected separately. Whole fruits at 3 and 7 days after pollination (DAP) were used. Only pericarps from fruits at 15 DAP, 25 DAP, mature green (MG), breaker (Br), or mature stage were collected and used. Three replicates of each sample were analyzed. For each replicate, 2–3 leaves, 5–6 flowers, 12–15 ovaries, and 6–12 fruits were collected.

Total RNA was extracted from the organs or tissues with TRIzol reagent (Invitrogen, USA), according to the manufacturer’s protocol. The cDNA was synthesized in a reaction of 20 μl with 1 μg RNA by using a PrimeScript RT reagent kit (Takara Bio, Japan). Quantitative real-time PCR (qRT–PCR) analysis was performed in a 20 μl reaction volume comprising 10 μl of SYBR Premix ExTaq (Takara Bio, Japan), 2 μl of template cDNA, 0.8 μl of each primer (10 μM), 0.4 μl of ROX II dye (×50), and 6 μl of sterilized water. The amplification was assayed on the StepOnePlus Real-Time PCR System (Applied Biosystems, USA). The expression of each gene was normalized to an internal reference *SlUBQ3*, which is a common reference gene in tomato and shows relatively stable expression under various conditions (Løvdal and Lillo, 2009). Relative expression levels were calculated by the standard curve method. The primers for *SlUBQ3* and *SlMYB3Rs* are shown in Supplementary Table S1.

### Vector construction and plant transformation

Three 20 bp target sequences followed by a trinucleotide (5ʹ-NGG-3ʹ) protospacer adjacent motif were designed by using CRISPRdirect (https://crispr.dbcls.jp/); the target sequences (gRNA1, gRNA2, and gRNA3) are shown in Supplementary Table S1. The backbone of the CRISPR/Cas9 vector was described in Kawaguchi *et al* (2021). The target sequences for *SlMYB3R3* were inserted between the *AtU6* promoter and the single guide RNA (sgRNA) scaffold to construct the target-specific sgRNA cassette by a PCR method. Then, the target sgRNA cassettes were linearized and inserted into the *Asc*I and *Mlu*I restriction sites of the CRISPR/Cas9 vector by the restriction cloning method to produce the multiplex-editing plasmid. The recombined CRISPR construct used in this study contained three sgRNA expression cassettes. The construct was electroporated into *Agrobacterium tumefaciens* strain GV2260.

Tomato ‘Suzukoma’ seeds were surface-sterilized for 2 min with 70% ethanol and 10 min with 1% (v/v) sodium hypochlorite solution, followed by rinsing with sterile water for 1 min five times. After sterilization, the seeds were kept in sterile water under darkness overnight and germinated in a plant box with Murashige and Skoog medium with 1.5% (w/v) sucrose and 0.3% (w/v) gelrite at 25 °C under 16 h light/8 h dark. After 10 d, the cotyledons were cut and infected by the *A. tumefaciens* strain GV2260 harboring the CRISPR/Cas9 vector. The transformation of tomato cotyledons was performed according to the modified method of Amemiya *et al.* (2006). T_0_ transformants were first screened in Murashige and Skoog medium with 100 mg/l Kanamycin. The DNA was extracted from leaves of WT plants and transformants according to the method of Edwards *et al.* (1991). The transgenic plants were confirmed by PCR amplification with the primers Cas9F/R, and the genome-edited plants were confirmed using the primers SlMYB3R3_EdF/R. The PCR products from the edited plants were used for sequencing to identify the targeted mutagenesis using the primer SlMYB3R3_Seq. All the primers mentioned above are shown in Supplementary Table S1. During genetic transformation of tomato, tetraploid plants are more likely to be obtained. Given the differences in morphology and physiology between tetraploid and diploid plants, only diploid plants can be used for further phenotypic evaluation. Therefore, the ploidy of the T_0_ generation was checked by Ploidy Analyzer PA type (Partec, Germany), and the diploid plants were self-crossed to obtain the T_1_ generation with homozygous mutations in the *SlMYB3R3* gene.

### Histological observations

Flower buds and ovaries at 0 DAP were analyzed. Flower buds and ovaries were vacuum-infiltrated in FAA fixation solution (formaldehyde:acetic acid:50% ethanol, 5:5:90) for 10 min three times and then kept in the FAA solution overnight. Subsequently, the samples were dehydrated in a concentration gradient of ethanol solutions (50%, 60%, 70%, 80%, 90%, 99.5%, 100%), cleared with Histo-Clear, and embedded in paraffin. The specimens were sectioned at a thickness of 12 μm by a rotary microtome (Leica RM2135, Germany) and then spread on to glass slides. The specimens were immersed in Histo-Clear to remove the paraffin and then rehydrated with an ethanol gradient series (100%, 90%, 70%, 50%, 30%). The sections were stained with 0.1% toluidine blue solution and mounted on the slides in Eukitt mounting medium (O. Kindler/Orsatec, Germany) under a cover glass. The slides were examined using an Olympus BX60 microscope equipped with a DP70 digital camera system (Olympus, Japan). The flower buds and whole ovaries were observed under a ×4 objective lens, and the details of the placenta and septum of the ovary were observed under a ×20 objective lens.

### Fruit phenotype statistics

For calculation of fruit indices, 27 matured fruits (from five independent plants) of the WT and lines #128, #151, #170, and #171, respectively, and 14 fruits (from three independent plants) for line #94 were used; the length and width of the fruits were measured by using a vernier caliper. The regions referred to as the placenta and septum are shown in Supplementary Fig. S1; the placenta and septum lengths of the fruits were measured by using ImageJ software (https://imagej.nih.gov/ij/). The cell number and size were measured as described by Kobayashi *et al.* (2015), and the average cell sizes of the columella and septum were calculated using ImageJ based on paraffin sections observed under the ×20 objective lens. The septum and ovary length were also measured using ImageJ based on the transverse and longitudinal paraffin sections of the ovary; at least five tissue sections for each line were used for the calculations. The cell numbers were calculated using data for the columella/septum and cell areas. Throughout this study, group differences were analyzed by using Dunnett’s test.

### RNA-Seq library preparation and sequencing

Flowers at the bloom stage were sampled and immediately frozen in liquid nitrogen. Three biological replicates for the WT and line #170 were used for RNA sequencing (RNA-Seq), and each biological replicate contained at least five blooming flowers. Total RNA was extracted with TRIzol reagent according to the manufacturer’s protocol. RNA quality was measured using a total RNA Quantus Fluorometer and QuantiFluor RNA system (Promega, USA). RNA quantity was assessed using a Qsep100 DNA Fragment Analyzer and RNA R1 Cartridge (BiOptic, Taiwan). The libraries were prepared using an MGIEasy RNA Directional Library Prep Set (MGI, China) according to the manufacturer’s instructions. The concentration of the libraries was measured by Qubit and a dsDNA HS Assay Kit (Thermo Fisher Scientific, USA), and the quantity was checked using Fragment Analyzer and a dsDNA 915 Reagent Kit (Advanced Analytical Technologies, UK). Paired-end reads (150 bp) were generated on the DNBSEQ-G400 at Bioengineering Lab. Co. (Kanagawa, Japan).

### RNA-Seq data analysis

Clean reads were filtered by removing low-quality bases with value <20, short reads with length ≤30 bp, and adaptor sequences using Sickle (Joshi and Fass, 2011). Then, the clean reads were aligned to the tomato reference genome ITAG4.0 (ftp://ftp.solgenomics.net/tomato_genome/assembly/build_4.00/) by hisat2 (ver.2.1.0) (Kim *et al.*, 2015). The reads were counted by featureCounts (ver.1.6.3) (Liao *et al.*, 2014). After normalization by iDEGES (Sun *et al.*, 2013), the differentially expressed genes (DEGs) were identified by the R package edgeR (Robinson *et al.*, 2010). The gene expression data were shown as reads per kilobase of transcript per million mapped reads (RPKM).

### Promoter *cis*-acting element analysis

The 3 kb upstream sequences of the transcription site for each gene were defined as promoter regions. The promoter sequences of the DEGs were retrieved in Fasta format from the tomato reference genome ITAG4.0 and then submitted to the PlantCARE database (http://bioinformatics.psb.ugent.be/webtools/plantcare/html/) for MSA *cis*-acting element analysis.

### Yeast one-hybrid assay

Three copies of the MSA motif (5ʹ-TCCAACGGT-3ʹ) were inserted into the *Hin*dIII site of the pAbAi vector by the In-fusion cloning method. The pMSA-AbAi vector was linearized with the *Bst*BI restriction enzyme, then the linearized pMSA-AbAi vector was transformed into Y1Hgold stain to generate the bait yeast strain. The coding sequence of *SlMYB3R3* was amplified using PrimeSTAR® Max DNA Polymerase (Takara Bio, Japan) and cloned into the pGADT7 vector. The pGADT7-*SlMYB3R3* and AD-empty (pGADT7 empty) vector were transformed into the MSA bait yeast strain. The AD-Rec-p53/p53 promoter was a positive control and AD-empty/MSA motif was a negative control. The three combinations of yeast cells were grown in synthetic defined medium without uracil and leucine (SD/-Ura/-Leu), with or without 200 ng ml^–1^ aureobasidin A (AbA), for 3 d at 30 °C.

### Accession numbers

The accession numbers of MYB3Rs were as follows: NtmybA1 (AB056122.1), NtmybA2 (AB056123.1), NtmybB (AB056124.1), AtMYB3R1 (AT4G32730.2), AtMYB3R2 (AT4G00540.1), AtMYB3R3 (AT3G09370.2), AtMYB3R4 (AT5G11510.1), AtMYB3R5 (AT5G02320.1), OsMYB3R-1 (ABA96851.2), OsMYB3R-2 (Q0JHU7.1), OsMYB3R-3 (XP_015637435.1), OsMYB3R-4 (XP_015625869.1), TaMYB3R1 (ADO32617.1), ZmMYB3R (XP_008672992.1), *Drosophila melanogaster* Myb (X05939.1), human B-Myb (X13293.1), human A-Myb (X13294.1), and human c-Myb (M15024.1). The accession number for the tomato reference gene *SlUBQ3* is X58253.1.

## Results

### Identification and characterization of *SlMYB3R*s in tomato

**Fig. 1.**
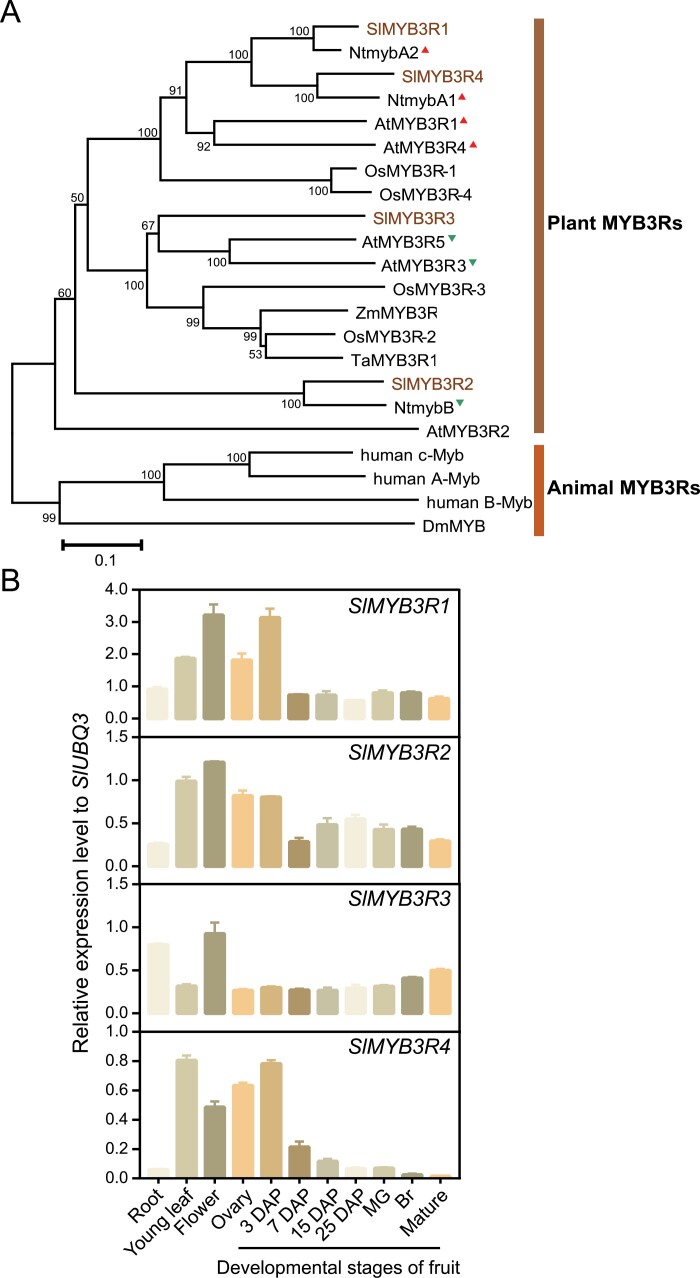
Phylogenetic and expression pattern analyses of *SlMYB3Rs*. (A) Phylogenetic tree of MYB3Rs of tomato (*Solanum lycopersicum*), Arabidopsis (*Arabidopsis thaliana*), tobacco (*Nicotiana tabacum*), rice (*Oryza sativa*), maize (*Zea mays*), wheat (*Triticum aestivum*), Drosophila (*Drosophila melanogaster*) and humans (*Homo sapiens*). Tomato SlMYB3Rs are shown in brown text. Red triangles indicate transcriptional activators in the corresponding plant species; green triangles indicate suppressors. The scale bar represents 0.1 substitutions per site. (B) Gene expression of *SlMYB3R*s in *S. lycopersicum* ‘Suzukoma’. The expression level is shown as the value relative to the expression of *SlUBQ3*. Data are the means ±SD from three replicates of independent samples. DAP, days after pollination; MG, mature green; Br, breaker.

Four *MYB3R* genes were identified in the genome of *S. lycopersicum* ‘Heinz’ and named *SlMYB3R1*, *SlMYB3R2*, *SlMYB3R3*, and *SlMYB3R4*. Amino acid alignment of *SlMYB3R*s with *MYB3R*s from other plant species, such as Arabidopsis and tobacco showed a high sequence similarity in the DNA-binding domain (Supplementary Fig. S2). The N-terminal domain of these MYB3R family members harbored three imperfect repeat sequences (R1, R2, and R3). Phylogenetic analysis of tomato *SlMYB3R*s with other associated *MYB3R*s showed that *SlMYB3R1* and *SlMYB3R4* clustered with the transcriptional activators *NtmybA2* and *NtmybA1*, respectively, whereas *SlMYB3R2* clustered with the repressor *NtmybB* (Fig. 1A). In contrast, *SlMYB3R3* was placed in a subclade with two Arabidopsis repressors (*MYB3R3* and *MYB3R5*). In addition, four animal *MYB3R*s formed a separate clade distant from the plant *MYB3R*s.

The tissue and temporal specificity of gene expression are strongly coupled to specific biological functions. To understand the possible functions of *SlMYB3R*s, qRT–PCR analysis using *S. lycopersicum* ‘Suzukoma’ was performed. *SlMYB3R1*, *SlMYB3R2*, and *SlMYB3R4* exhibited similar expression patterns (Fig. 1B); as such, these genes were specifically expressed in the leaves, flowers, ovaries, and fruit at 3 DAP but showed low expression in the fruit at later developmental stages. *SlMYB3R1* and *SlMYB3R2* exhibited mildly fluctuating expression in the fruit during the ripening process, whereas *SlMYB3R4* expression gradually decreased with fruit growth. *SlMYB3R3* expression was highest in flowers, followed by roots. *SlMYB3R3* expression in the fruit was lower than that in flowers; however, its transcript levels gradually increased with fruit ripening. The expression levels of these four *MYB3R*s in various organs and tissues of *S. lycopersicum* ‘Heinz’ and its wild relative *Solanum pimpinellifolium*, obtained from the Tomato eFP Browser, are presented in Supplementary Fig. S3. The expression patterns of *MYB3R*s in ‘Suzukoma’ were generally consistent with those in ‘Heinz’ and *S. pimpinellifolium*.

Based on the results of the gene expression patterns and phylogenetic analyses, we focused our subsequent work on *SlMYB3R3*, which was placed in a clade with two repressors in Arabidopsis, *MYB3R3/5*, and showed overall higher gene expression than the other three *SlMYB3R*s in all organs or tissues of both cultivated and wild tomato varieties.

### Generation of *SlMYB3R3* knockout lines using CRISPR/Cas9 in ‘Suzukoma’

**Fig. 2.**
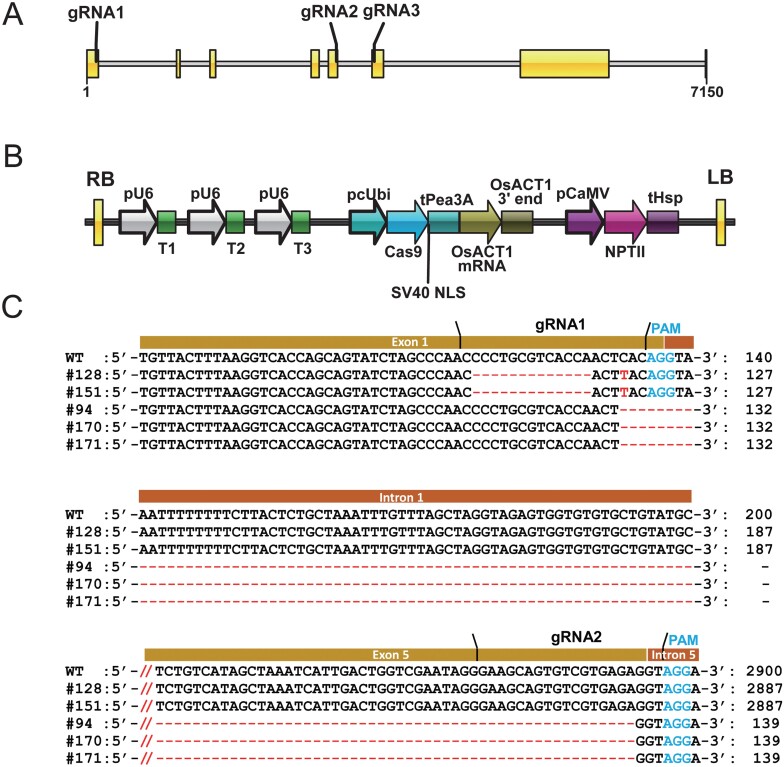
Targeted mutagenesis of *SlMYB3R3* in tomato by the CRISPR/Cas9 system. (A) Gene structure of *SlMYB3R3* and the locations of three target sequences (gRNA1, gRNA2, and gRNA3) for the CRISPR/Cas9 system. (B) Schematic of the CRISPR/Cas9 vector with key regulatory elements. pU6, Arabidopsis U6-26 promoter; pcUbi, parsley ubiquitin promoter; Cas9, *Streptococcus pyogenes* CRISPR-associated endonuclease 9; SV40 NLS, SV40 nuclear localization signal; tPea3A, pea rbcS3A terminator; OsACT1 mRNA, rice *actin 1* gene; OsACT1 3ʹ end, rice *actin 1* 3ʹ end sequence; pCaMV, cauliflower mosaic virus 35S promoter; NPT II, kanamycin resistance gene; tHsp, heat shock protein terminator. RB and LB are T-DNA border sequences. (C) Genomic sequences around gRNA1 and gRNA2 of the WT and the *slmyb3r3* mutants. The deleted nucleotides are represented by red dashes, and nucleotide substitutions are shown in red text. The target sequences (gRNA1 and gRNA2), exons, and introns of *SlMYB3R3* are labeled. // represents an omission of 2641 nucleotides. The numbers indicate nucleotide number, counting from the start codons.

To determine the regulatory functions of *SlMYB3R3* during the development of ‘Suzukoma’, we generated *slmyb3r3* mutants by targeted mutagenesis using the CRISPR/Cas9 system. *SlMYB3R3* contained eight exons and the genomic fragment of *SlMYB3R3* was 7150 bp in length. Three target sequences (gRNA1, gRNA2, and gRNA3) flanked by the protospacer adjacent motif were located in the first, fifth, and sixth exons of *SlMYB3R3*, respectively. Subsequently, the three target sequences were inserted downstream of the *AtU6-26* promoter (Fig. 2A, B; Supplementary Table S1). Targeted mutagenesis of *SlMYB3R3* in the T_1_ generation was confirmed by direct sequencing of *SlMYB3R3* PCR products amplified from genomic DNA.

Five independent T_1_ progenies with homozygous mutations at the *SlMYB3R3* locus were selected. The mutated target sites in each line are shown in Fig. 2C. Two types of cleaved polymorphisms were identified among the five homozygotes. Specifically, two lines (#128 and #151) exhibited a 13 bp deletion and a single nucleotide substitution at the gRNA1 target locus, and three lines (#94, #170, and #171) exhibited a 2761 bp deletion, which resulted from successful cleavage at the gRNA1 and gRNA2 target loci. Both types of sequence polymorphisms led to frameshift mutations in *SlMYB3R3*, which ultimately resulted in non-functional proteins. Therefore, studies were performed using these five mutants.

### Morphology of reproductive organs in *slmyb3r3* mutants

**Fig. 3.**
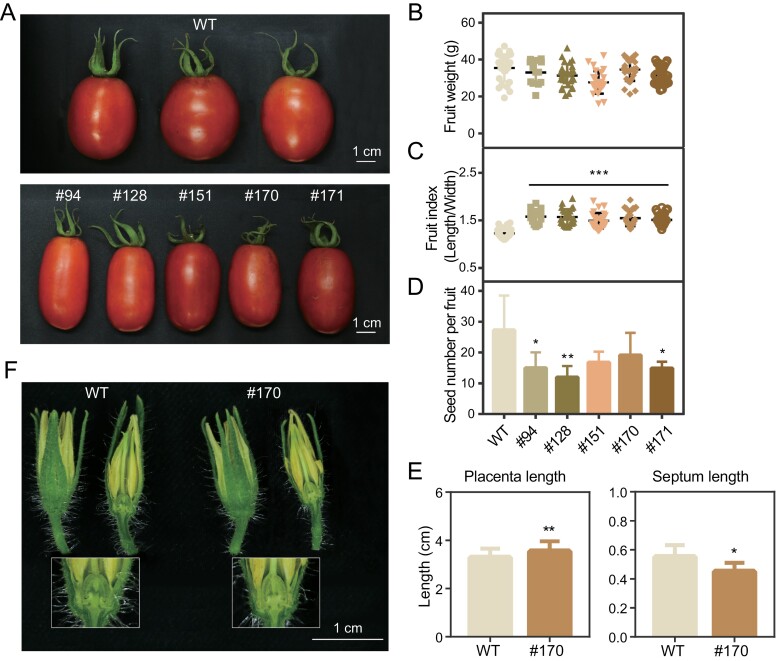
Phenotypic features of reproductive organs from the WT and the *slmyb3r3* mutants (#94, #128, #151, #170, and #171) at the mature stage. (A) Fruit morphologies of the WT and the *slmyb3r3* mutants. (B) Weight distribution of ripe fruits. (C) Fruit shape index (length/width). (D) Number of seeds per fruit. (E) Placenta and septum length of ripe fruits. (F) Whole and longitudinally sectioned flower buds of the WT and *slmyb3r3* mutant #170. Data are means ±SD. Significant differences are indicated with asterisks: ***P<0.001, **P<0.01, *P<0.05.

The *slmyb3r3* mutants grew normally in the greenhouse and showed no alteration in plant stature, fruit set, or vegetative organ architecture compared with the WT. However, aberrant development of reproductive organs was observed. The morphology of the WT fruit was characterized by an elliptical shape at maturity, whereas the five *slmyb3r3* mutants in the T_1_ generation had a much more elongated fruit shape (Fig. 3A). The fruit shape index (length/width) of the *slmyb3r3* mutants was significantly higher (28.7% in #94, 27.8% in #128, 21.7% in #151, 25.9% in #170, and 23.2% in #171) than that of the WT (1.23), but the weight distribution of the fruits of the *slmyb3r3* mutant did not differ significantly from that of WT fruits (Fig. 3B, C). The seed numbers in #94, #128, and #171 were lower than those in the WT; however, no significant alterations were observed in samples from lines #151 and #170 (Fig. 3D).

All five *slmyb3r3* mutants exhibited similar phenotypes, and line #170 was selected for further evaluation of *SlMYB3R3* function. From the proximal–distal and medial–lateral axes of the fruit at maturity, we found that the placenta was longer, while the septum was shorter, in mutant #170 than in the WT (Fig. 3E; Supplementary Fig. S1). To determine when the elongated fruit formed, we selected flower buds and observed the ovaries. The appearance of flower buds did not differ significantly between WT and #170 plants. However, examination of longitudinal sections of the flowers revealed that the ovary of #170 was longer than that of the WT (Fig. 3F).

### Histological observations of ovaries in the *slmyb3r3* mutants

**Fig. 4.**
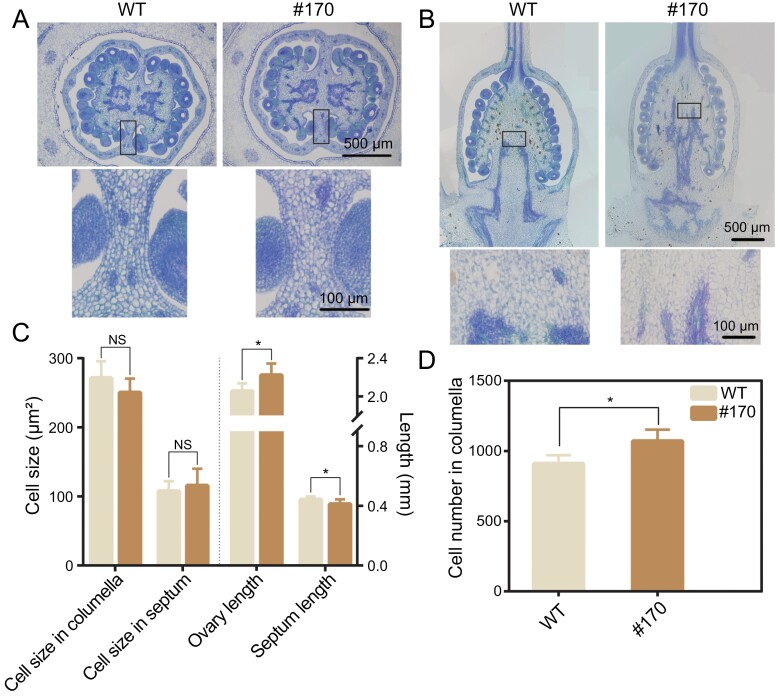
Histological observations of the placenta and septum from the WT and the *slmyb3r3* mutant #170 at 0 DAP. (A) Transverse paraffin sections of the ovary. The upper panels show the overall appearance of the whole ovary under a ×4 objective lens and the lower panels show an enlarged view of the septum (indicated by the black box in the upper panels) under a ×20 objective lens. (B) Longitudinal paraffin section of the ovary. The upper panels show the overall appearance of the whole ovary under a ×4 objective lens and the lower panels show an enlarged view of the placenta (indicated by the black box in the upper panels) under a ×20 objective lens. (C) Cell size of the columella and septum subregions and length of the ovary and septum based on measurements made on the longitudinal and transverse paraffin sections. (D) Cell number of the columella based on measurements made on the longitudinal sections. Five independent ovary sections were used for the measurements of the WT and #170, respectively. Significant differences are indicated with asterisks: ***P<0.001, **P<0.01, *P<0.05. NS, not significant.

As an elongated ovary was observed before anthesis, we isolated the ovaries from flower buds and obtained paraffin-embedded sections. Quantitative analysis of ovary sections showed that the ovary was significantly longer in #170 than in the WT, and the septum in #170 was shorter than that in WT (Fig. 4A, B). Closer examination of cellular parameters within the columella in the longitudinal direction and the septum in the transverse direction showed no significant difference in cell size between the WT and #170, but the cell number in the longitudinal direction was significantly increased in #170 (Fig. 4C, D). These findings show that the elongated fruit shape develops early and is caused by altered cell division, with an increased cell number in the longitudinal direction.

### External stimuli from surrounding organs affect ovary growth

**Fig. 5.**
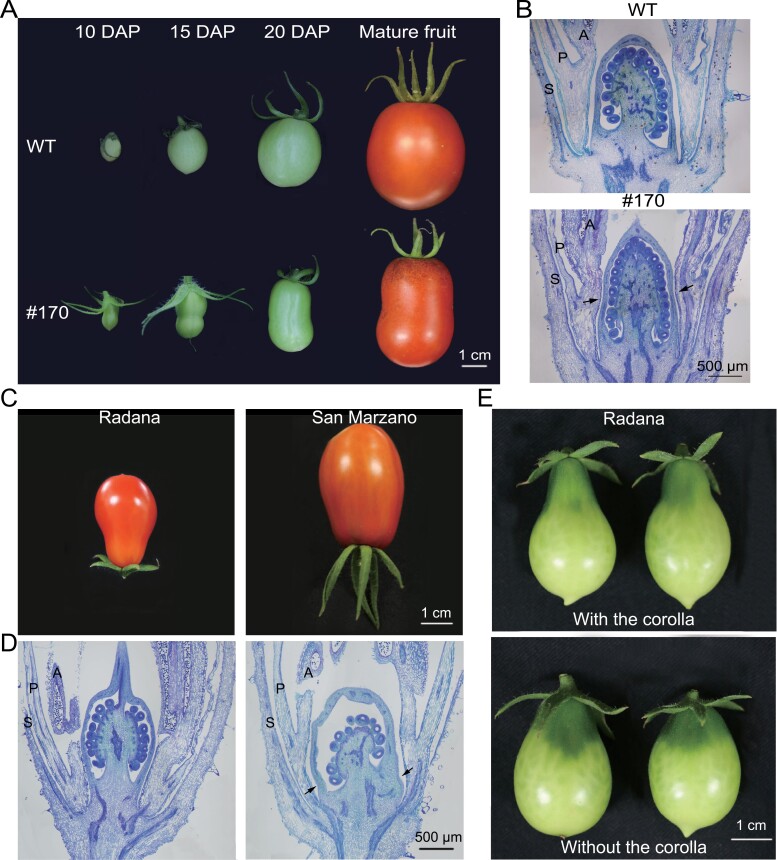
Histological and phenotypic observations of fruits. (A) Fruit morphologies of the WT and the *slmyb3r3* mutant #170 at 10, 15, and 20 DAP, and the mature stage. (B) Longitudinal paraffin sections of flower buds at 0 DAP. Arrows indicate the constriction in the ovary wall of #170. (C) Morphologies of mature fruits of ‘Radana’ and ‘San Marzano’. (D) Longitudinal paraffin sections of flower buds of ‘Radana’ and ‘San Marzano’ at 0 DAP. (E) Fruit morphologies of ‘Radana’ at the mature green stage, showing fruits that had developed in the presence or absence of the corolla. S, sepal; P, petal; A, anther.

Detailed examination of tomato fruit morphology at maturity revealed that some fruits (26.9% in #94, 38.95% in #128, 40.66% in #151, 28.52% in #171, and 34.9% in #170) had a peanut-like shape (Fig. 5A; Supplementary Table S2). The peanut-like shape became obvious at 10–18 DAP. At later stages, this shape was counteracted by the enlarged fruit size, and it became less obvious with fruit ripening. Examination of paraffin-embedded sections of representative flower buds revealed a slight constriction in the ovary wall of #170, which transformed the ovary into a peanut-like shape (constrictions are indicated by arrows in Fig. 5B). Consistent with the elongated fruit shape of the *slmyb3r3* mutants (Fig. 3A), the peanut-like fruit shape was linked to ovary development.

To investigate the development of tomato fruit with classical shapes, we selected two tomato cultivars, ‘Radana’ and ‘San Marzano’. These two cultivars exhibit specific but different shapes at maturity and are representative of typical shapes in development studies. In ‘Radana’, the ripened fruit showed a markedly elongated neck at the proximal end. In ‘San Marzano’, the ripened fruit was long and bumpy in shape, and had a constriction near the proximal end at maturity (Fig. 5C). Longitudinal sections of the flower buds of the two cultivars were examined. The ovary of ‘Radana’ showed a conspicuous elongated neck, similar to ‘Yellow Pear’ as described in the literature (Liu *et al.*, 2002; Wu *et al.*, 2018). Likewise, a constriction close to the proximal end of the ovary was observed in ‘San Marzano’; the uneven surface of the ovary was highly similar to the mature fruit (arrows in Fig. 5D). These results indicate that the *slmyb3r3* mutants and existing cultivars with specific fruit shapes undergo similar fruit-shaping processes, with the formation of the specific shape of the fruit being synchronized with the onset of the ovary meristem.

In addition to genetic regulation by *SlMYB3R3*, the surrounding organs also play an important role in fruit morphogenesis as external regulators. Corolla removal alleviates the marked constriction in the proximal part of the fruit, resulting in an oval fruit (Ku *et al.*, 1999). To determine the effects of floral organs on fruit shape, we removed the petals and fused stamen cones from ‘Radana’ following pollination. Differences in fruit morphology were observed at the MG stage. The fruit with the corolla maintained a narrower neck at the proximal end, whereas in fruit lacking the corolla the neck was less narrow and the fruit showed a moderately rounded end (Fig. 5E). We performed a similar experiment with *slmyb3r3* mutants and found that the proportion of peanut-shaped fruits reduced to ~20% in #170 when the corolla was removed following pollination. We hypothesized that in addition to *SlMYB3R3* function, an external stimulus, which appeared to be mechanical force from the surrounding aberrant floral organs, resulted in constriction of the ovary wall and led to the peanut-like fruit shape.

### DEGs in floral organs between the WT and *slmyb3r3* mutants

Transcriptome analysis was performed to elucidate the function of *SlMYB3R3* in mediating fruit elongation. Given the elongated ovary shape observed at anthesis, we selected blooming flowers of WT and the #170 mutant for RNA-Seq analysis. In total, 21 606 genes were expressed in at least one library, according to the RPKM ≥1 threshold. Setting a *q*-value (i.e. false discovery rate) of <0.05 as the criterion for significance, 270 genes were identified as DEGs between #170 and WT. Among them, 232 genes were up-regulated and 38 genes were down-regulated in #170 compared with the WT (Supplementary Table S3).

Many cell-cycle-related genes specifically expressed in the G2/M phase were up-regulated in the *slmyb3r3* mutant. For instance, eight cyclin genes, including *CYCA1* (*Solyc11g005090.2.1*), *CYCB1* (*Solyc06g073610.3.1* and *Solyc10g080950.2.1*), and *CYCB2* (*Solyc02g082820.3.1*, *Solyc04g082430.3.1*, *Solyc12g094600.3.1*, and *Solyc03g032190.3.1*), and one B2-type CDK gene (*CDKB2*, *Solyc04g082840.4.1*) were up-regulated in the *slmyb3r3* mutants (Table 1). The cell division cycle 20.2 (*CDC20.2*) gene, which is the cofactor of the anaphase-promoting complex or cyclosome (APC/C) that regulates cell cycle progression (Hein and Nilsson, 2016), was also positively regulated.

**Table 1.** List of representative differentially expressed genes between the *slmyb3r3* mutant #170 and the WT

Gene ID	Gene annotation	Log_2_(*slmyb3r3*/WT)
*Solyc03g096870.3.1*	Cell division cycle 20.2, cofactor of APC complex-like	3.34
*Solyc02g082820.3.1*	Cyclin B2	3.56
*Solyc04g082430.3.1*	CyclinB2_4	3.32
*Solyc12g094600.3.1*	CyclinB2_5	9.89
*Solyc06g072830.4.1*	Cell division cycle 20.2, cofactor of APC complex-like	2.81
*Solyc05g014370.3.1*	Mitotic spindle checkpoint protein MAD2	2.01
*Solyc03g007130.4.1*	65-kDa microtubule-associated protein 3	2.35
*Solyc06g053760.3.1*	Syntaxin-related protein KNOLLE	1.84
*Solyc08g005420.3.1*	Cell division cycle 20.2, cofactor of APC complex-like	2.39
*Solyc09g097860.4.1*	Kinesin-like protein KIN12B	1.85
*Solyc04g082900.4.1*	Mitotic spindle checkpoint protein BUBR1	2.16
*Solyc07g042560.4.1*	Kinesin-like protein NACK1	2.96
*Solyc11g017130.2.1*	FHA domain-containing protein PS1	2.43
*Solyc10g080950.2.1*	CyclinB1_2	2.31
*Solyc01g079750.3.1*	MAP kinase kinase kinase 4	1.36
*Solyc12g095870.3.1*	Aurora kinase A-A	1.06
*Solyc06g073610.3.1*	Cyclin B1	1.53
*Solyc10g078330.2.1*	B-type cyclin	1.82
*Solyc04g082840.4.1*	B2-type cyclin dependent kinase	0.93
*Solyc03g032190.3.1*	CyclinB2_7	1.5
*Solyc03g121760.3.1*	SUN-like protein 11	1.92
*Solyc08g062940.4.1*	SUN-like protein 22	1.32
*Solyc01g099270.2.1*	Microtubule-binding protein TANGLED1	1.51
*Solyc01g098980.4.1*	MAP kinase kinase kinase 8	0.77
*Solyc11g005090.2.1*	Cyclin A1	0.6
*Solyc12g098630.2.1*	Kinesin-like protein KIN12B	1.03
*Solyc01g091380.4.1*	65-kDa microtubule-associated protein 9	0.89
*Solyc01g107630.4.1*	Cell division cycle protein 123 homolog	0.61
*Solyc11g072630.2.1*	Mitogen-activated protein kinase 4	0.87

Moreover, genes related to cytokinesis were up-regulated in the *slmyb3r3* mutant. The genes *solyc11g017130.2.1* (encoding the FHA domain-containing protein PS1), *solyc12g095870.3.1* (encoding Aurora kinase A-A), and six targeting proteins for Xklp2 (TPX2) family members were positively regulated. These genes likely function in spindle organization during mitosis (D’Erfurth *et al.*, 2008; Vos *et al.*, 2008; Petrovská *et al.*, 2012). *Solyc05g014370.3.1* and *solyc04g082900.4.1*, which encode the spindle checkpoint proteins MAD2 and BUBR1, respectively, were also up-regulated in the *slmyb3r3* mutant. These two proteins ensure correct chromosomal attachment to the microtubule spindle by kinetochores for proper segregation of sister chromatids (Bolanos-Garcia and Blundell, 2011; Lara-Gonzalez *et al.*, 2012).

The gene *solyc01g099270.2.1* encodes the microtubule-binding protein TANGLED1 (TAN1), whose counterpart in maize controls cell plate orientation and cell cycle progression (Cleary and Smith, 1998; Smith *et al.*, 2001; Martinez *et al.*, 2017; Mir *et al.*, 2018). *Solyc06g053760.3.1*, an ortholog of the Arabidopsis gene *KNOLLE*, encodes a syntaxin-related protein with a specific function in cytokinesis (Lukowitz *et al.*, 1996; Boutté *et al.*, 2010). *Solyc07g042560.4.1* encodes the kinesin-like protein NACK1. In tobacco, NACK1 is an activator of the MAP kinase kinase kinase (MAPKKK) NPK1, which is necessary for cell plate expansion (Nishihama *et al.*, 2002; Vavrdová *et al.*, 2019). Another kinesin-like protein, KIN12B, is essential for microtubule organization and cell plate development (Lee *et al.*, 2007; Müller and Livanos, 2019). These genes were up-regulated in the *slmyb3r3* mutant (Table 1). In addition, two *SUN*-like genes were significantly up-regulated in the *slmyb3r3* mutant. Overall, the majority of the DEGs were identified as cell-cycle- or cytokinesis-related genes, and they showed significantly increased expression in the #170 mutant.

### SlMYB3R3 regulates gene expression by directly binding to MSA motifs

In a pioneering study, MYB3Rs were shown to activate or inhibit the expression of cell-cycle-related genes by specifically binding to the *cis*-acting element MSA (Ito *et al.*, 2001; Haga *et al.*, 2011; Kobayashi *et al.*, 2015). Therefore, we analyzed the MSA motifs in the promoter regions of the 270 DEGs. A total of 46 DEGs containing at least one MSA motif were identified, the majority of which were cell-cycle- or cytokinesis-related genes (Supplementary Fig. S4; Supplementary Table S4). For example, two B-type cyclin genes, *CYCB1* and *CYCB2*, are indispensable CDK cofactors. Four kinesin protein genes, of which three encode unknown proteins and one encodes KIN12B, were identified. Genes encoding two CDC20.2 proteins, spindle checkpoint protein BUBR1, FHA domain-containing protein PS1, syntaxin-related protein KNOLLE, and SUN-like protein 11 also had MSA motifs in their promoters.

**Fig. 6.**
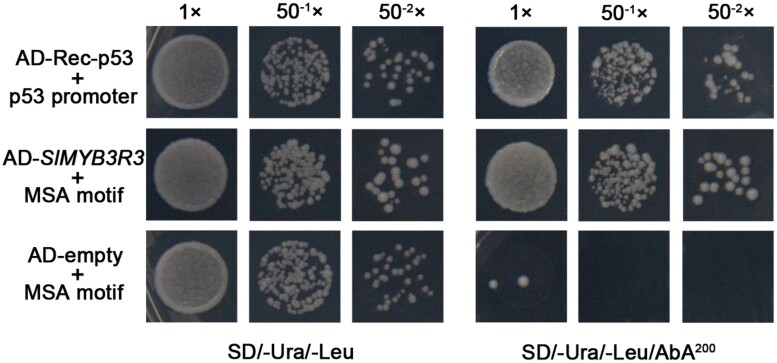
Yeast one-hybrid assay showing the interactions between SlMYB3R3 and the MSA motif. The AD-Rec-p53+p53 promoter is a positive control and the AD-empty+MSA motif is a negative control. Co-transformed yeast cells (diluted and undiluted) were grown on SD/-Ura/-Leu medium with or without 200 ng ml^–1^ AbA.

To investigate the relationships between SlMYB3R3 and the genes containing MSA motifs in their promoters, we performed a yeast one-hybrid assay. The MSA motif was used to construct the yeast bait strain and *SlMYB3R3* was cloned into the pGADT7 vector. The AD-Rec-p53/p53 promoter was used as the positive control. All yeast cells grew normally in SD/-Ura/-Leu medium; however, the yeast strain transformed with AD-empty failed to grow in the SD/-Ura/-Leu medium with 200 ng ml^–1^ AbA (Fig. 6). These results show that SlMYB3R3 directly binds to MSA motifs in the promoters.

## Discussion

### SlMYB3R3 functions as a suppressor of cell cycle genes in an MSA-dependent manner

The MYB3Rs constitute a small subfamily within the large MYB family and play conserved roles in both plants and animals. They are primarily linked to cell cycle regulation, particularly in the G2/M phase (Kranz *et al.*, 2000; Stracke *et al.*, 2001). Tobacco NtmybA1/A2 and its counterparts in Arabidopsis (MYB3R1/4) activate the expression of G2/M-specific genes by recognizing the MSA motif in the promoters (Ito *et al.*, 2001; Ito, 2005; Kato *et al.*, 2009). Microarray analysis of *myb3r1*/*4* mutants showed that multiple cell cycle genes specific to the G2/M phase were down-regulated, and MSA motifs were significantly enriched in the promoters of these genes (Haga *et al*., 2007, 2011). Conversely, NtmybB in tobacco and MYB3R3/5 in Arabidopsis function as suppressors, and cell cycle genes such as *CYCB1*, *CYCB2*, *CDKB2*, *NACK1*, and *KNOLLE* are up-regulated in *myb3r1/3/5* mutants (Kobayashi *et al.*, 2015; Chen *et al.*, 2017). *SlMYB3R3* is an ortholog of the Arabidopsis *MYB3R3/5*. In the *slmyb3r3* mutant #170, most DEGs (232 of 270) were up-regulated. Up-regulation was associated with cell cycle genes, especially genes specific to the G2/M phases, such as *CYCB2*, *CYCB1*, *CDKB2*, and *CDC20.2*, and some M-related genes, such as *TAN1*, *NACK1*, and *KNOLLE* (Table 1). The up-regulated genes in the present study largely overlapped with those of *myb3r*s mutants in a previous study. Furthermore, many up-regulated genes contained MSA-like motifs in their promoters and were directly repressed by SlMYB3R3. Therefore, SlMYB3R3 functions as a suppressor of cell-cycle-related genes by binding to their MSA motifs.

### A cohort of cytokinetic DEGs is crucial for fruit elongation

The Arabidopsis *myb3r1/4* and *myb3r1/3/5* mutants showed irregularly oriented division planes and disorganized cell patterns during embryo development, and multiple cytokinesis-related genes were differentially expressed. Irregular cell division is largely ascribed to cytokinetic genes, particularly microtubule-associated proteins (Haga *et al*., 2007, 2011; Kobayashi *et al.*, 2015). In addition, we found more evidence that the well-identified fruit regulators OVATE and SUN appear to alter cell division by interacting with microtubules during the cytokinesis process (Lazzaro *et al.*, 2018). OVATE likely regulates microtubule arrays by physically interacting with members of the TONNEAU1-recruiting motif (TRM) family. TRMs modulate the assembly of the TON1–TRM–PP2A complex and direct the complex towards microtubules (Liu *et al.*, 2002; Drevensek *et al.*, 2012; Wu *et al.*, 2018). IQD family members in plants, including SUN, regulate organ shapes. The SUN orthologs CsSUN in cucumber and ClFS1 in watermelon have been identified as candidate regulators of fruit shape (Pan *et al.*, 2017; Dou *et al.*, 2018). The SUN ortholog IQD12 in Arabidopsis is localized to microtubules. Overexpression of IQD in Arabidopsis alters organ morphology and microtubule orientation (Bürstenbinder *et al.*, 2017). Overexpression of *SUN* in tomato results in an elongated fruit shape as a consequence of SUN interacting with the cytoskeleton (Lazzaro *et al.*, 2018). Overexpression of the *SUN*-like gene *OsIQD14* in rice results in longer and narrower grains via microtubule reorientation (Yang *et al.*, 2020). In the present study, many microtubule-associated genes showed differential expression in mutants when compared with the WT. These included the genes encoding FHA domain-containing protein PS1 (*Solyc11g017130.2.1*), aurora kinase A-A (*Solyc12g095870.3.1*), spindle checkpoint proteins MAD2 and BUBR1 (*Solyc05g014370.3.1* and *solyc04g082900.4.1*), microtubule-binding protein TAN1 (*Solyc01g099270.2.1*), kinesin-like proteins KIN12B and NACK1 (*Solyc12g098630.2.1* and *Solyc07g042560.4.1*), and some TPX2s (Supplementary Table S3). In addition, SUN-like proteins 11 and 22 showed differential expression between the *slmyb3r3* mutant and the WT (Supplementary Table S4). These proteins are closely related to Arabidopsis IQD6/7/8, which regulate microtubule dynamics and organization (Huang *et al.*, 2013; Liang *et al.*, 2018). Furthermore, the MSA motif was identified in the promoter of SUN-like protein 11 and in many microtubule-associated genes (Supplementary Table S4; Supplementary Fig. S4). The effects of these DEGs on microtubule activity may be responsible for the altered cell division patterns.

### SlMYB3R3 may target genes up-regulated in the *slmyb3r3* mutant but unrelated to the cell cycle

In addition to regulating cell-cycle-related genes, MYB3Rs are involved in stress resistance by regulating stress-related genes. OsMYB3R-2 activates multiple cold-responsive genes and increases the stress tolerance of rice by sensing freezing, drought, and salt stress signals (Dai *et al.*, 2007). Overexpression of *AcMYB3R* in kiwifruit enhances plant resistance to drought and salinity stress (Zhang *et al.*, 2019). Furthermore, macroscopic differences in plant organ development were observed in the *myb3r*s mutants. These mutants exhibit increased seed, embryo, and leaf size (Kobayashi *et al.*, 2015). *myb3r1/4* mutants showed reduced internodes, immature seeds with a ball shape, one or more cotyledons than the number in the WT, and occasionally fused cotyledons. Several down-regulated genes that were unrelated to the cell cycle may be involved in the pleiotropic phenotypes, which cannot be explained by the roles of cell-cycle-related genes in *myb3r1/4* mutants (Haga *et al.*, 2011). In the *slmyb3r3* mutants, we also found that some of the flowers (~30%) exhibited more slender petals than WT flowers (Supplementary Fig. S5). One possible explanation is that SlMYB3R3 regulates the expression of genes unrelated to the cell cycle. This explanation also helps in understanding the growth of the unexpected peanut-like tomato shapes. Stimuli from the abnormal corolla influenced the formation of peanut-like fruit shapes. Mechanical force is considered a crucial stimulus for peanut-like fruit shaping.

The E2F *cis*-element (5ʹ-WTTSSCSS-3ʹ) is also one of the target motifs of MYB3R proteins, and is present in the promoters of most E2F TFs that are associated with DNA replication and S phase entry (Vandepoele *et al.*, 2005; Kobayashi *et al.*, 2015). In the present study, only 46 of the 270 DEGs harbored MSA in their promoters. Another alternative explanation is that SlMYB3R3 targets motifs other than the MSA motif, such as the E2F-like motif. We found that six DEGs had the E2F motif in their promoters (Supplementary Table S5).

### *SlMYB3R3* is a potential target gene for breeding to enrich various agronomic traits

The goal of crop development is to improve crop quality, appearance, and nutritive value, and make crops more attractive to consumers. Many well-identified genes have been used as targets to improve crop quality (Liu *et al.*, 2021). By applying the CRISPR/Cas9 system to edit these target genes, fruits or vegetables with certain phenotypes can be obtained. Some MYB TFs, functioning as causal genes to alter the color of plants with a rapidly increased content of flavonoids, carotenoids, or other metabolites, have been applied for crop breeding to increase the health benefits of these plants (Allan and Espley, 2018). Researchers have produced yellow tomato fruit by knocking out the phytoene synthase *PSY1* gene and pink tomato fruit by knocking out the flavonoid-related TF *MYB12* (Filler Hayut *et al.*, 2017; Yang *et al.*, 2019). By targeting *ANT1*, researchers have created purple tomatoes rich in anthocyanins that benefit human health (Vu *et al.*, 2020). In addition to color modification, other traits are considered to drive crop improvement. Knock out of the gene encoding pectin-degrading enzyme (*PL*) in tomato results in firmer fruit and prolonged shelf life (D. Wang *et al*., 2019). Aromas have been introduced into unscented rice varieties to produce aromatic rice by modulating the fragrance gene *OsBADH2* using CRISPR/Cas9 (Ashokkumar *et al.*, 2020). Editing of the TRM gene *TaGW7* in wheat produces grains with increased width and weight (W. Wang *et al*., 2019). The fruit shape regulators *OVATE*, *fas*, and *lc* have been used to improve the genetic diversity of tomato through genome editing (Rodríguez-Leal *et al.*, 2017; Zsögön *et al.*, 2018). A long fruit shape shows higher efficiency in transportation and packaging than a round fruit. As such, *SlMYB3R3*, the elongated fruit shape regulator identified in this study, is a potential target gene for modifying the appearance of tomato. Combined with molecular breeding technologies, transgene-free tomato fruits with an elongated shape could be produced.

## Supplementary data

The following supplementary data are available at *JXB* online.

Table S1. The primer sequences used in this study and target sequences of *SlMYB3R3* for the CRISPR/Cas9 system.

Table S2. The percentage of pear-like-shaped fruit in the mutants.

Table S3. All the DEGs between the *slmyb3r3* mutant and WT.

Table S4. List of DEGs harboring the MSA motif in their promoter regions.

Table S5. List of DEGs harboring the E2F-like motif in their promoter regions.

Fig. S1. Longitudinal and transverse sections of mature fruits.

Fig. S2. Alignment of the amino acid sequences of SlMYB3Rs and typical MYB3Rs from other plant species.

Fig. S3. Gene expression of *SlMYB3Rs* in *S. lycopersicum* ‘Heinz’ and *S. pimpinellifolium*.

Fig. S4. Schematic of MSA *cis*-acting elements residing in the promoter regions of the 46 genes.

Fig. S5. Morphologies of floral organs of WT and the *slmyb3r3* mutant #170.

erac352_suppl_Supplementary_Figures

erac352_suppl_Supplementary_Tables

## Data Availability

The sequence data of the WT and #170 have been deposited in the DNA Data Bank of Japan (DDBJ) database (BioProject ID PRJDB11281, BioSample IDs SAMD00283693–SAMD00283698). All other data supporting the findings of this study are available within the paper and within its supplementary data published online.

## Abbreviations

AbA: aureobasidin A
Br: breaker
CDK: cyclin-dependent kinase
CYC: cyclin
DAP: day after pollination
M: mitosis
DEG: differentially expressed gene
MG: mature green
MSA: mitosis-specific activator
qRT–PCR: quantitative real-time PCR
sgRNA: single guide RNA
TF: transcription factor
WT: wild type
