# Supplementary material for: Genome editing of SlMYB3R3, a cell cycle transcription factor gene of tomato, induces elongated fruit shape
Source: J Exp Bot. 2022 Sep 7;73(22):7312–25. doi: 10.1093/jxb/erac352 (PMC9730800; doi:10.1093/jxb/erac352)
Supplement: erac352_suppl_Supplementary_Figures [file erac352_suppl_supplementary_figures.pdf]

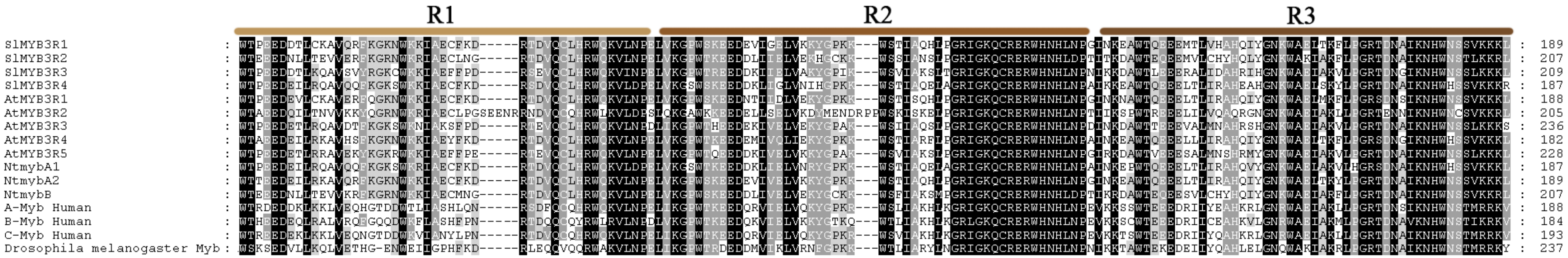

Fig. S1 Alignment of amino acid sequences of SlMYB3Rs and typical MYB3Rs from other plant species. The conserved R1, R2, and R3 DNA binding domains are indicated. Identical amino acids are shaded in black, and similar amino acids are shaded in gray.

Gene name	Gene locus	Description	***S. lycopersicum* 'Heinz'**										***S. pimpinellifolium***			
			Bud	Flower	Leaf	Root	1cm fruits	2cm fruits	3cm fruits	MG	Breaker (B)	B + 10days	Immature fruits	Breaker (B)	B + 5days	Leaf
SlMYB3R1	Solyc08g068320	Transcription factor myb (AHRD V1 *** Q5QT32_ORYSA); contains Interpro domain(s) IPR015495 Myb transcription factor	2.6	1.37	1.53	6.22	5	2.61	1.53	0.97	0.36	0.28	1.6	0.92	0.24	5.29
SlMYB3R2	Solyc08g080580	Polypeptide feature inferred from GFF3 feature	1.7	0.94	1.29	1.67	1.69	1.96	1.54	1.77	1.02	0.73	2.89	3.59	2.07	1.17
SlMYB3R3	Solyc09g010820	MYB transcription factor (AHRD V1 **** Q6R032_ARATH); contains Interpro domain(s) IPR015495 Myb transcription factor	5.29	7.57	6.11	8.75	5.25	5.03	6.45	6.15	6.11	7.28	9.78	10.5	9.52	2.78
SlMYB3R4	Solyc11g071300	Myb (AHRD V1 *** Q948S6_TOBAC); contains Interpro domain(s) IPR015495 Myb transcription factor	1.38	0.48	0.33	2.05	2.53	1.12	0.61	0.22	0.03	0	0.64	0.22	0.09	2.51

Fig. S2 Gene expressions of *SlMYB3Rs* in *S. lycopersicum* 'Heinz' and *S. pimpinellifolium*. The gene expression data are derived from RNA sequencing and are downloaded from Tomato eFP browser (http://bar.utoronto.ca/efp2/Tomato/Tomato_eFPBrowser2.html). The expression values are RPKM-normalised.

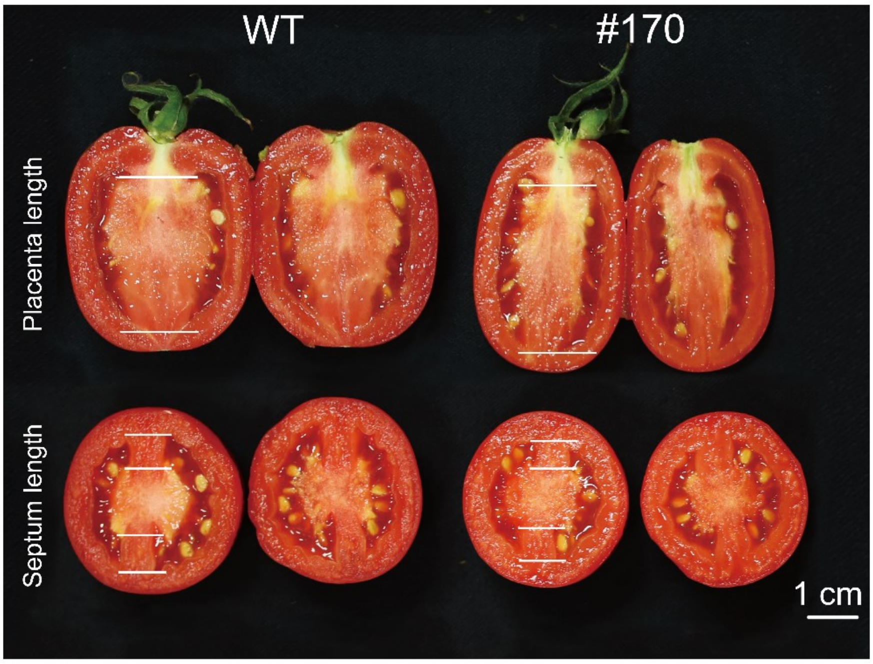

Fig. S3 Longitudinal and transversal sections of mature fruits. Placenta and septum are marked by the white lines, the distances between the two lines are defined as placenta and septum length, respectively.

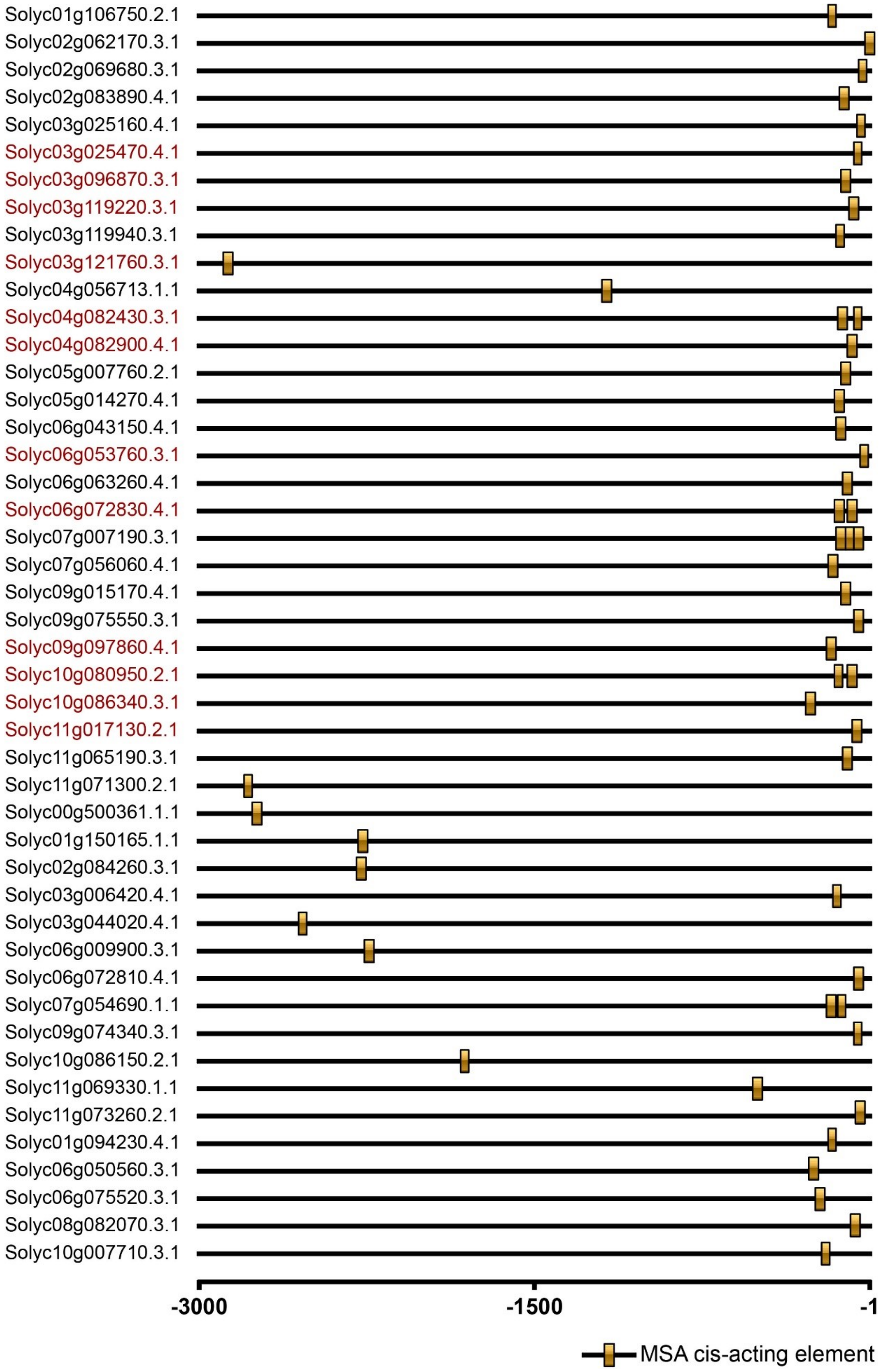

Fig. S4 Schematic of MSA cis-acting elements residing in the promoter regions of the 46 genes. The gene id marked in dark red suggests this gene is annotated as a cell cycle-related gene.

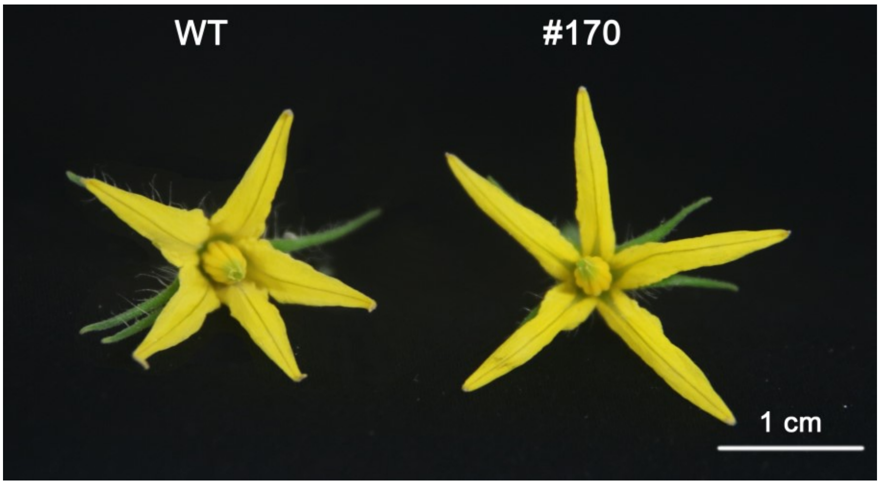

Fig. S5 Morphologies of floral organs of WT and the *slmyb3r3* mutant #170.
